# Eliminating Contamination in Umbilical Cord Blood Culture Sampling for Early-Onset Neonatal Sepsis

**DOI:** 10.3389/fped.2021.794710

**Published:** 2021-12-20

**Authors:** Vilmaris Quinones Cardona, Vanessa Lowery, David Cooperberg, Endla K. Anday, Alison J. Carey

**Affiliations:** ^1^Department of Pediatrics, St Christopher's Hospital for Children, Philadelphia, PA, United States; ^2^Department of Pediatrics, Drexel University College of Medicine, Philadelphia, PA, United States; ^3^Department of Pediatrics, Sidney Kimmel College of Medicine, Thomas Jefferson University, Philadelphia, PA, United States; ^4^Department of Microbiology and Immunology, Drexel University College of Medicine, Philadelphia, PA, United States

**Keywords:** early onset sepsis (EOS), umbilical cord blood (UCB), quality improvement (QI), peripheral blood culture (PBC), umbilical cord blood culture (UCBC)

## Abstract

**Introduction:** Despite the advantages of umbilical cord blood culture (UCBC) use for diagnosis of early onset sepsis (EOS), contamination rates have deterred neonatologists from its widespread use. We aimed to implement UCBC collection in a level III neonatal intensive care unit (NICU) and apply quality improvement (QI) methods to reduce contamination in the diagnosis of early onset sepsis.

**Methods:** Single-center implementation study utilizing quality improvement methodology to achieve 0% contamination rate in UCBC samples using the Plan-Do-Study-Act (PDSA) model for improvement. UCBC was obtained in conjunction with peripheral blood cultures (PBC) in neonates admitted to the NICU due to maternal chorioamnionitis. Maternal and neonatal characteristics between clinical sepsis and asymptomatic groups were compared. Process, outcome, and balancing measures were monitored.

**Results:** Eighty-two UCBC samples were collected in addition to peripheral blood culture from neonates admitted due to maternal chorioamnionitis. Ten (12%) neonates had a diagnosis of clinical sepsis. All PBCs were negative and 5 UCBCs were positive in the study period. After 2 PDSA cycles, there was special cause variation with improvement in the percent of contaminated samples from 7.3 to 0%. There was no change in antibiotic duration among asymptomatic neonates.

**Conclusions:** Implementation of UCBC for the diagnosis of EOS in term infants is feasible and contamination can be minimized with the implementation of a core team of trained providers and a proper sterile technique without increasing antibiotic duration.

## Introduction

Neonatal sepsis is a significant cause of morbidity and mortality. Since the use of intrapartum antibiotic prophylaxis (IAP), the incidence of early onset sepsis (EOS) is higher among preterm, very low birth weight infants as compared to term infants. However, the burden of disease among term infants remains substantial, with 60% requiring neonatal intensive care for respiratory distress and/or cardiovascular support (1, 2). Peripheral blood culture (PBC) is the gold standard for detection of neonatal sepsis; however, a minimum volume of 1 mL of blood is necessary when a single pediatric blood culture bottle is used (3). Blood culture sensitivity decreases by 10–40% when 0.5 mL is inoculated instead of 1 mL and so inadequate volume submitted for blood cultures can lead to under diagnosis of culture positive sepsis (4, 5).

Umbilical cord blood is an underutilized resource in the care of neonates. Cord blood is used for blood type, antibody screen, and chromosome analysis (6). Utilizing cord blood in high-risk infants to obtain blood cultures has been shown to increase the etiological diagnosis of sepsis (7). Umbilical cord blood culture (UCBC) has a sensitivity between 80 and 100% and specificity between 91.4 and 94.9% when compared to PBC for the diagnosis of sepsis in high-risk newborns (8). The procedure is painless and technically less challenging when compared to peripheral vein or artery puncture in a term or preterm neonate and has been shown as a possible alternative for peripherally obtained infant blood in sepsis evaluation (9, 10).

Despite these advantages, UCBCs have not been routinely adapted as standard of care due to concern of higher contamination rates without clinical correlation (11). False positive blood cultures can lead to clinical uncertainty and increase antibiotic use. Implementation of a strict sterile sampling technique in peripherally obtained neonatal blood cultures has been shown to reduce contamination (12). We aimed to implement UCBC collection in a level III neonatal intensive care unit (NICU) and apply quality improvement (QI) methods to reduce contamination with the goal of replacing PBC in the diagnosis of EOS.

## Methods

This was a single-center implementation study utilizing quality improvement methodology in an 18-bed Level III academic urban delivery hospital NICU in Philadelphia, Pennsylvania, USA. The study aim was to achieve 0% contamination rate in UCBC samples using the Plan-Do-Study-Act (PDSA) model for improvement in a Level III NICU by December 2018. The study population included all inborn neonates ≥ 37 weeks' gestation who were admitted to the NICU with suspected sepsis due to maternal chorioamnionitis. Maternal chorioamnionitis was defined as maternal fever (>38.0°C) and one of the following: uterine fundal tenderness, maternal tachycardia (>100 beats/min), fetal tachycardia (>160 beats/min), and purulent or foul amniotic fluid. Exclusion criteria were: <37 weeks' gestation, no history of maternal chorioamnionitis, or not admitted to NICU. This population was deliberately chosen to ease the implementation of UCBC sampling as these neonates were usually clinically stable and, at the time of the study, routinely admitted to the NICU.

This prospective study was conducted between March 2017 and December 2018. UCBC collection was implemented in addition to the standard PBC collection workflow for all patients ≥ 37 weeks' gestation admitted to the NICU for sepsis evaluation in the setting of maternal chorioamnionitis. Given UCBC is not standard of care in EOS, this implementation study was designed to collect both UCBC and PBC samples to ensure standard of care was being followed with PBCs while assessing the provider concerns of UCBC contamination and inferior performance in the management of EOS. Quality improvement methodology was planned to assess and mitigate UCBC contamination with the goal of replacing PBCs with UCBCs based on study results.

Prospective data collection included gestational age, weight, perinatal risk factors, culture negative vs. culture positive sepsis, results of PBCs and UCBCs, duration of antibiotics, and if repeat blood or cerebral spinal fluid (CSF) cultures were performed. The effectiveness of implementation processes was evaluated using PDSA cycles in accordance with quality improvement methodology. This study was approved by the Drexel Institutional Review Board (IRB) and verbal consent was required from the mother prior to sample processing.

Umbilical cord blood cultures were not obtained in our NICU before this study. The medical provider team who routinely obtained PBCs in the NICU included residents, fellows, neonatal nurse practitioners (NNPs), and attending neonatologists. All infants with maternal chorioamnionitis at the time of the study were routinely admitted to the NICU for assessment, continuous monitoring, PBC, and were started on intravenous (IV) ampicillin and gentamicin. Duration of antibiotic therapy was at the discretion of the neonatologist based on symptomatology, laboratory, and PBC results. Asymptomatic infants with negative blood cultures were transferred to the mother-baby unit at 24–48 h for continued monitoring and completion of 48 h of antibiotics. Symptomatic infants remained in the NICU for monitoring and IV antibiotic therapy. Delayed cord clamping was routinely practiced. All placentas were routinely sent to pathology for evaluation and results were available ~5–7 business days after delivery.

## Interventions

### Implement a Standardized Sterile Blood Culture Collection Process and Technique

The UCBC collection sampling technique implemented was a hybrid between our local sterile technique utilized for PBC samples and some elements used in a UCBC collection description available online (13). A clamped segment of the umbilical cord was obtained by obstetricians and given to the neonatal provider. To allow for adequate comparison to PBC, the UCBC sampling technique introduced in March 2017 also included donning hat, mask, and sterile gloves. The cord was wiped 3 times with povidone-iodine and allowed to dry. Using a sterile 22-gauge needle (bevel-down) and syringe, at least 1 mL of blood was collected from the umbilical artery or vein. Once blood was collected, the top of the blood culture bottle was cleaned with alcohol, a new sterile needle was connected to the syringe, and the blood was transferred to the blood culture bottle. Samples were sent to the laboratory within 1 h of collection. In April 2018, the sample collection technique was modified to include drying the umbilical cord segment before initiating the sterile collection process to enhance effectiveness of betadine and optimize sterility.

To improve the collection process, UCBC sample collection kits were stocked in the delivery carts and included hat, mask, sterile gloves, 1 pack of 3 povidone-iodine sticks, 1 alcohol pad, 2 sterile 22-gauge needles, 1 sterile 3 mL syringe, a blood culture bottle, and a disposable changing pad. Fellows and NNPs were added to the study protocol in December 2017 and new fellows in July 2018 to maximize the number of providers with consenting privileges and increase collection of UCBC samples.

### Communication

To maximize enrollment, verbal and written signage was provided to labor and delivery (L&D) teams, neonatal providers, and nursing. This study was also discussed as the learning point of the week during L&D rounds. These rounds, which took place every day at 8 a.m. and 8 p.m., included the neonatology and obstetrical teams to discuss patients in labor, post-operative delivery complications, high-risk antepartum cases, and a learning point for the week. Early NICU notification at the time of diagnosis of maternal chorioamnionitis, the utility of UCBC, and the need for a clamped cord segment for an UCBC was highlighted in March 2017. Day and night champions aided in sustaining long-term awareness of this initiative and reminded fellows and NNPs that a patient was eligible for a UCBC. Additionally, with the launch of this study, the microbiology team notified a designated study team member in real time of all positive UCBCs.

### Education

Prior to implementation of UCBC sampling in March 2017, a 20-min educational session was provided to neonatal fellows, NNPs, and a select group of attendings on the utility of UCBC and the UCBC sterile sampling technique. A nursing-specific session was provided to increase awareness and served as an added layer to remind providers if a patient was eligible for UCBC collection. In September 2017, one-on-one education was performed with four new fellows on UCBC sterile sample collection. In September 2017 and February 2018, one-on-one refreshers of the sterile UCBC sampling technique was done with the providers that had obtained a sample which was considered a contaminant. In April 2018, training on the newly modified sample collection technique which included drying the umbilical cord before application of betadine was reviewed with fellows, NNPs, and attendings. This training on the modified technique was repeated in July 2018 with new incoming first-year fellows.

## Measures

To evaluate effectiveness of implementation, we measured the percentage of UCBC and PBC samples obtained from eligible patients during the study period. Eligible patients and collection compliance were monitored through review of neonatal admission log and electronic health record (EHR). The outcome measure was the contamination rate of UCBC samples. Contamination was defined as a positive UCBC with >1 organism or a positive UCBC without a clinical diagnosis of sepsis. Process measures included communication from the laboratory with the study team with positive UCBC results and availability of UCBC collection kits by neonatal provider report.

UCBC results in the EHR could cause uncertainty in medical management, inadvertently prolong the duration of antibiotics, or trigger further diagnostic studies in asymptomatic cases. Therefore, the duration of antibiotics and repeat diagnostic studies were also monitored as balancing measures.

## Analysis

The number of eligible admissions, neonatal and maternal characteristics, UCBC, PBC, and placental pathology results were collected to assess the impact of the interventions. Neonatal and maternal characteristics between clinical sepsis and asymptomatic groups were compared using student *t*-test for continuous data and Fisher's exact test for categorical data. Data was analyzed with statistical package for social sciences (SPSS) software version 24. The outcome, process, and balancing measures were displayed using statistical process control (SPC) P and X bar/S charts, respectively. QI-Macros 2020 was used to analyze and generate SPC charts. Centerlines and 3- delta control limits were applied when at least 8 consecutive points were above or below the center line, one or more data points fell beyond the control limits, or six consecutive points trended in either direction (14, 15). Centerlines were adjusted based on detection of special cause signal (16).

## Results

A total of 82 out of 97 (84.5%) eligible neonates had both UCBC samples and PBC collected during the 22-month period. All UCBC samples were included and there were no lost samples. Reasons for missed opportunities to collect UCBC samples included: provider forgot to obtain UCBC (*n* = 7), inability to obtain consent (*n* = 4), unavailable UCBC kit in the delivery cart (*n* = 1), or post-natal diagnosis of chorioamnionitis after placenta was sent to pathology (*n* = 3).

A total of 10 neonates had a diagnosis of clinical sepsis and 72 were asymptomatic. There was no difference in maternal temperature, group B streptococcus status, exposure to intrapartum antibiotics, or evidence of placental chorioamnionitis among groups (Table 1). Neonatal characteristics were also similar between groups except for antibiotic duration which was longer in the clinical sepsis group with an average of 7.2 ± 2.1 vs. 2 days in the asymptomatic group. Among the 10 neonates with clinical sepsis, all had negative PBCs and two had single organism growth in UCBCs (*Escherichia coli* and *Streptococcus anginosus*, Table 2). Nine neonates with clinical sepsis had placental evidence of chorioamnionitis. Only one of the two with a single organism UCBC had confirmed placental chorioamnionitis (*Streptococcus anginosus*).

**Table 1:** Neonatal and maternal characteristics.

**Parameters**	**Clinical sepsis** **(***n*** = 10)**	**Asymptomatic** **(***n*** = 72)**	***p*** **-value**
Gestational age (mean weeks, SD)	40.1 ± 1	39.4 ± 1.1	0.19
Delivery type (C-section, %)	5 (50%)	26 (36%)	0.49
Weight (mean grams, SD)	3,704 ± 474	3,387 ± 458	0.07
Gender (male, %)	6 (60%)	43 (60%)	1
5-min Apgar score (mean, SD)	8.2 ± 1.2	8.7 ± 0.8	0.24
Maternal temperature (mean F, SD)	101.4 ± 0.9	101.2 ± 0.7	0.64
Prolonged rupture of membranes (>18 h, %)	4 (40%)	18 (25%)	0.45
Group B streptococcus positive (%)	2 (20%)	18 (25%)	1
Maternal intrapartum antibiotics (%)	10 (100%)	65 (90%)	0.59
Placental chorioamnionitis (%)	9 (90%)	44 (63%)*	0.152
Antibiotic duration (days, SD)	7.7 ± 2.1	2 ± 0	<0.001

* *2 placental pathology results not available*.

*F, fahrenheit; SD, standard deviation*.

**Table 2:** Positive umbilical cord blood culture results.

**Case**	**UCBC results**	**PBC results**	**Clinical sepsis**
2	*Enterococcus faecalis*	Negative	No
7	*Escherichia coli*	Negative	Yes
17	*Streptococcus mitis*	Negative	No
31	*Citrobacter freundi, Enterococcus faecalis, Staphylococcus hemolyticus*	Negative	No
41	*Streptococcus anginosus*	Negative	Yes

*UCBC, umbilical cord blood culture*.

**Figure 1:**
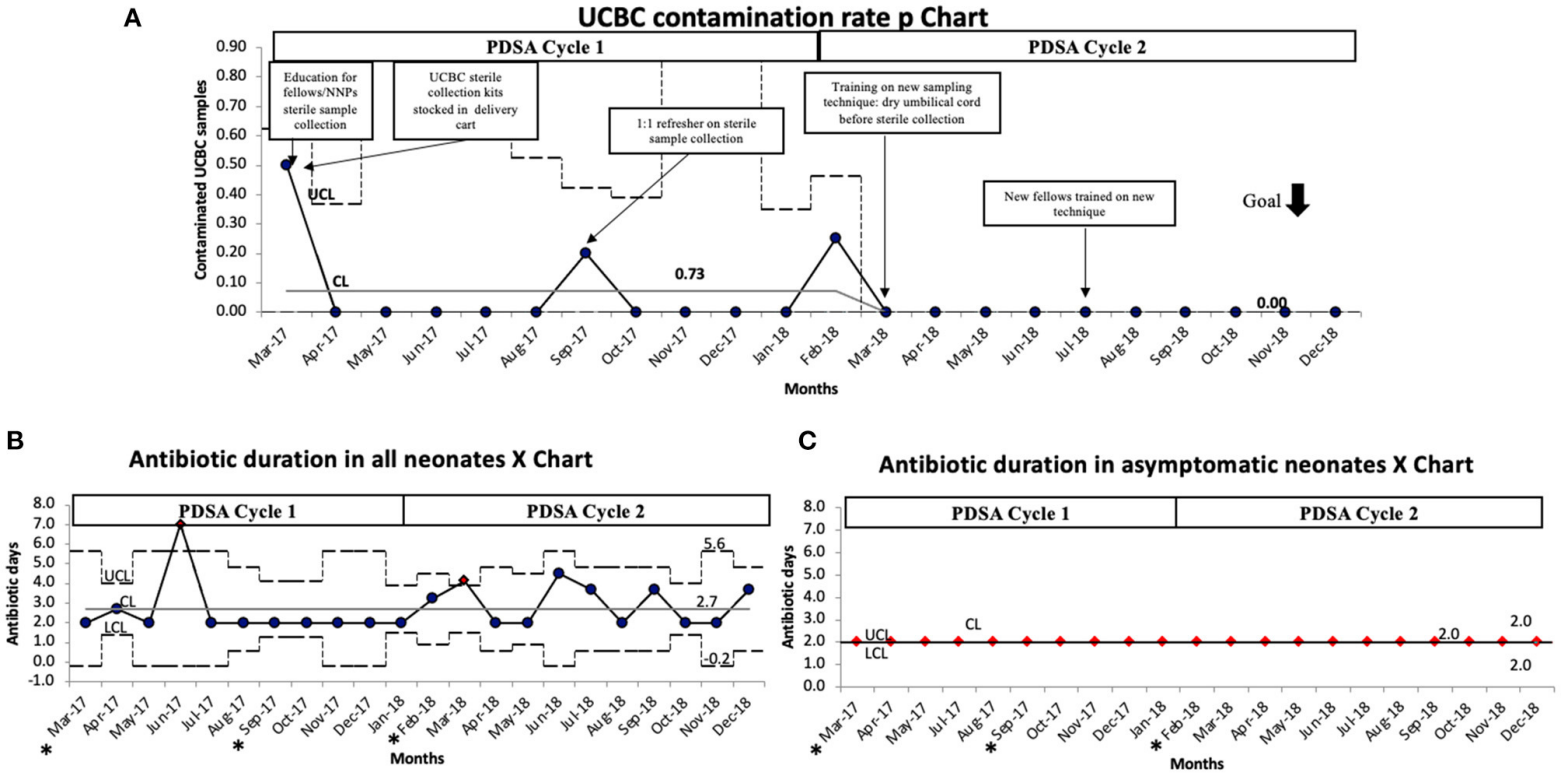
**(A)** UCBC contamination rate P chart demonstrating special cause variation with a reduction of contamination from 7.3 to 0%. **(B)** Antibiotic duration in all neonates P chart demonstrating variability but no change in the average duration of antibiotics during this initiative. **(C)** P chart showing antibiotic duration specifically in asymptomatic neonates did not change during the initiative. The black dotted line denotes upper and lower control limits, solid gray line denotes the center line, solid black line denotes average per month. CL, center line (mean); LCL, lower control limit; PDSA, plan do study act; UCL, upper control limit. *months with contaminated samples.

After sequential process changes, there was special cause variation, with 8 consecutive points below the center-line, in the percent of contaminated UCBC samples from 7.3 to 0% (Figure 1A). The organisms isolated in each UCBC are described in Table 2. One-on-one refreshers revealed failed sterile technique in 2 out of 3 contaminated samples (#2, #17, and #31). One sample (#17) was obtained without sterile technique by an untrained obstetrical nurse trying to assist the team while the neonatal provider performed resuscitation and the other (#31) also had break in sterility during collection by one of our fellows. The third contaminated sample (#2) was reported to be collected with sterile technique. This feedback prompted the modification of our sterile technique in April 2018 to include adequate drying of the umbilical cord segment before sterile sample collection. After this modification of the UCBC sterile collection process, we had 28 samples collected, 4 cases of clinical sepsis, and all patients had negative UCBC and PBC cultures (Table 3). The percent availability of UCBC collection kits throughout the study was 98.7%. The microbiology laboratory compliance with reporting all positive UCBC results to a study team member was 100%.

**Table 3:** Culture results in implementation compared to post-implementation periods.

	**Implementation period** **(***n*** = 54)**	**Post-implementation** **(***n*** = 28)**
Clinical sepsis, *n* (%)	6 (11)	4 (14)
Positive PBC, *n* (%)	0 (0)	0 (0)
Positive UCBC, *n* (%)	2 (8)	0 (0)
Contaminated UCBC, *n* (%)	3 (5.5%)	0 (0)

*PBC, peripheral blood culture; UCBC, umbilical cord blood culture*.

Balancing measures showed the average duration of antibiotics among all neonates was 2.7 days, with 2 points meeting special cause variation in June 2017 and March 2018 which were not related to contamination, rather 2 infants in each month treated for clinical sepsis (Figure 1B). This was further confirmed as the duration of antibiotics among asymptomatic patients remained unchanged at 2 days (Figure 1C). None of the cases with positive UCBC had repeat PBC or CSF cultures.

## Discussion

UCBC for diagnosis of EOS was successfully implemented in a level III NICU with QI process-driven reduction in contamination rates. While the overall UCBC contamination rate was 3.7% and aligns with published UCBC contamination rates of 1.5–12% (17, 18), key interventions to eliminate contamination included refining the sterile sample collection, drilling down on breaks in sterility with one-on-one sessions, and collaborating with experts in the field. Limiting the UCBC sterile sample collection to highly trained NNPs, fellows, and attendings improved the standardization of the collection technique. Although expanding the UCBC sample collection to neonatal or obstetrical nursing team was appealing, we determined that training a core group of providers would ensure utmost sterility in sample collection. To our knowledge, this is the first study that combines the implementation of UCBC for diagnosis of EOS and applies QI methodology to eliminate the main reason for UCBC underutilization which is contamination.

The practical applications of UCBCs include being a reliable diagnostic alternative for EOS with equivalent organisms identified compared to venous blood cultures (8, 19), allowing for necessary blood culture volumes to be obtained at no risk to the infant, and reducing the need for blood transfusions in preterm infants when umbilical cord blood is used for admission laboratories (6, 20–22). Here, once the sterile collection technique was refined, UCBC was non-inferior to PBC. Therefore, application of this UCBC sterile collection technique may serve as part of a multi-prong blood conservation strategy in this vulnerable population.

This initiative also highlights the challenges neonatologists face with clinical sepsis. There is hesitancy to trust negative neonatal blood cultures due to inadequate volume, when non-specific clinical signs are present, or when mothers receive adequate IAP (23). Infants born to mothers with adequate IAP are at lower risk for GBS EOS because IAP eliminates bacteria or reduces bacterial concentrations to ultralow concentrations, neither of which require additional antibiotic therapy (23). However, despite negative PBCs, adequate IAP, and resolution of symptoms while on antibiotics, some of these infants are treated for clinical or culture-negative sepsis. After implementation of the modified collection technique, UCBC and PBC results were congruent in the last 28 patients. Of these 28 patients, 14% had a diagnosis of clinical sepsis. Therefore, UCBC is a less invasive approach which yields equivalent results and conserves neonatal blood. This provides compelling evidence to change practice. Furthermore, volumes of 1 ml or greater can be easily obtained from the umbilical cord with a proper sterile technique. Therefore, this can provide clinicians reassurance that the UCBC results are accurate.

Our population was limited to a small convenience sample of term infants affected by maternal chorioamnionitis for ease of UCBC implementation and those for which parental consent was obtained to meet IRB standards. Although the management practices for asymptomatic neonates > 35 weeks born to mothers with chorioamnionitis continue to evolve (24–26), obtaining blood cultures in high-risk neonates with concern for sepsis is the standard of care. Despite these limitations, our results provide valuable data to initiate unit practice change for UCBC to be the sole blood culture required for EOS evaluation of term infants admitted to the NICU and/or share valuable lessons with those units who already have a UCBC program but are challenged by contamination. Hospital closure precluded tracking UCBC contamination rates beyond the study period which would have provided a larger sample in the post-implementation period and allowed further monitoring of quality of UCBC specimens. This is especially important in the setting of EOS which is already a rare event. The lack of positive PBCs in this study limited the accuracy assessment of UCBC results against positive PBCs which would have been a powerful comparison. However, this also correlates with published reports that EOS is a rare event. Additionally, given this study did not include preterm infants, validation of our UCBC sampling technique in preterm infants would be beneficial for widespread use as published data suggests UCBC sample collection is feasible in the setting of delayed cord clamping in this population (21).

Implementation of UCBC for the diagnosis of EOS in high-risk term infants is feasible and contamination can be minimized with the implementation of a core team of trained providers and a proper sterile technique. Collection of high-quality UCBCs, which pose no risk to the infant, can be achieved with equivalent results as the standard of care without adversely increasing antibiotic duration. Replication of this study at other sites including preterm infants would be beneficial. Furthermore, application of this sterile technique to draw UCBCs in preterm neonates may be a valuable strategy as part of a blood conservation bundle in this high-risk population.

## Data Availability Statement

The raw data supporting the conclusions of this article will be made available by the authors, without undue reservation.

## Ethics Statement

The studies involving human participants were reviewed and approved by Drexel University College of Medicine Institutional Review Board. Written informed consent from the participants' legal guardian/next of kin was not required to participate in this study in accordance with the national legislation and the institutional requirements.

## Author Contributions

VQC conceptualized and designed the study, collected and analyzed the data, drafted the initial manuscript, and reviewed and revised the manuscript. VL contributed to the design of the UCBC kits, collected samples and data, and reviewed and revised the manuscript. DC, EA, and AC contributed to study design, data analysis, critically reviewed and revised the manuscript for important intellectual content. All authors contributed to the article and approved the submitted version.

## Conflict of Interest

The authors declare that the research was conducted in the absence of any commercial or financial relationships that could be construed as a potential conflict of interest.

## Publisher's Note

All claims expressed in this article are solely those of the authors and do not necessarily represent those of their affiliated organizations, or those of the publisher, the editors and the reviewers. Any product that may be evaluated in this article, or claim that may be made by its manufacturer, is not guaranteed or endorsed by the publisher.
